# Association of DRG-based payment reform with inpatient costs and resource use among older adults: an interrupted time series study in a county-level referral hospital in China

**DOI:** 10.3389/fpubh.2026.1953054

**Published:** 2026-09-17

**Authors:** Jiacheng Zou, Shumin Wang, Jiali Shi, Jianhan Chen, Qing Guan, Shaohua Li

**Affiliations:** 1 School of Health Management, Anhui Medical University, Hefei, Anhui, China; 2 School of Public Health, Southeast University, Nanjing, Jiangsu, China; 3 Hefei Third People's Hospital, Hefei, Anhui, China; 4 YiXing People’s Hospital, Wuxi, Jiangsu, China; 5 The First Affiliated Hospital of Anhui Medical University, Hefei, Anhui, China; 6 The Fourth Affiliated Hospital of Anhui Medical University, Hefei, Anhui, China; 7 Anhui Provincial Institute of Healthcare Security, Anhui Medical University, Hefei, Anhui, China

**Keywords:** China, DRG-based payment, inpatient costs, interrupted time series, older adults

## Abstract

**Background:**

China has rapidly expanded its diagnosis-related group (DRG)-based payment reform; however, evidence primarily focuses on urban pilots or specific disease cohorts. There is limited understanding of how DRG implementation affects out-of-pocket (OOP) spending, cost composition, and resource utilization among older adults treated in county-level referral hospitals.

**Methods:**

We conducted a retrospective interrupted time series study utilizing administrative inpatient data from a tertiary county-level referral hospital in eastern China. From July 1, 2021, to July 31, 2025, we identified 154,744 admissions among adults aged ≥60. The outcomes assessed included total costs, out-of-pocket costs, drug costs, material costs, examination/laboratory costs, and length of stay (LOS). The primary analysis used admission-level segmented regression with standard errors clustered by calendar month. Supplementary analyses used monthly aggregated models with Newey-West standard errors and exploratory disease-group heterogeneity analyses.

**Results:**

In the adjusted admission-level interrupted time series models, the implementation of DRG was associated with an immediate reduction in OOP costs (*β*_2_ = −1360.617 CNY) and a more pronounced negative trend post-implementation (*β*_3_ = −106.705 CNY per month). LOS experienced an immediate increase (*β*_2_ = 0.831 days) and continued to rise in comparison to the pre-implementation trend (*β*_3_ = 0.077 days per month). Total-cost findings differed by the analytical level: no statistically significant immediate or slope change was observed in the admission-level model (β₂ = −84.683 CNY; β₃ = 34.844 CNY/month), whereas the monthly aggregated model showed an immediate decrease (β₂ = −851.064 CNY) followed by an upward post-intervention slope change (β₃ = 75.090 CNY/month). Changes in total costs and most cost components were not consistent across different model specifications. Monthly aggregated analyses indicated directionally similar results for OOP costs and LOS. Disease-group analyses revealed that reductions in OOP costs were more substantial for neoplasms, digestive diseases, and circulatory diseases, while increases in LOS were primarily observed in certain circulatory and respiratory conditions.

**Conclusion:**

Among older adult inpatients in a county-level referral hospital in China, DRG-based payment reform was associated with lower patient OOP but longer hospital stays. Its effects on total spending and cost composition were less consistent and varied across disease groups.

## Introduction

Population aging and sustained growth in health expenditure have intensified pressure on health systems to enhance efficiency without compromising access or quality (1, 2). In China, inpatient care continues to be a significant contributor to medical spending, and the long-standing reliance on fee-for-service payment has been linked to cost escalation and incentives for overprovision of services (3, 4). Consequently, prospective provider payment reform has emerged as a central policy tool. Among these reforms, Diagnosis-Related Group (DRG)-based payment is particularly influential, as it reimburses hospitals based on case mix rather than the volume of individual services delivered (5, 6).

International evidence suggests that DRG payment systems can influence hospital behavior, resource utilization, and quality of care; however, the direction and magnitude of these effects vary across different settings (7–9). In China, the reform of DRG payment has rapidly transitioned from local pilot programs to a national implementation within the health insurance framework (10, 11). Nevertheless, the empirical literature on this topic remains inconsistent. A systematic review of studies conducted in China revealed mixed outcomes concerning total expenditure, out-of-pocket (OOP) payments, quality, and equity, with considerable heterogeneity in research methods and definitions of outcomes (12). Subsequent studies from regions such as Beijing, Shanghai, and Anhui have documented reductions in expenditure or expenditure variation in certain contexts, while changes in length of stay (LOS) and quality have been limited or inconsistent in others (13–18).

These mixed findings align with the economic principles underlying DRG payment systems. By transferring a portion of financial risk from payers to providers, DRG payments can promote standardization, reduce low-value inputs, and limit service intensity (5, 12, 19). Concurrently, they may lead to adaptations in coding, substitutions in services, changes in discharge behavior, or selective restructuring of care. Consequently, total costs, OOP burdens for patients, cost composition, and LOS do not necessarily move in tandem. This asynchronous response may be particularly significant for older adults, whose care often involves multimorbidity, frailty, recurrent admissions, and more complex discharge planning (20–23).

Four evidence gaps are particularly significant. First, much of the existing evidence is derived from pilot cities, tertiary urban hospitals, or selected disease cohorts, while county-level referral hospitals and county medical alliance hubs have received less attention, despite their central role in the implementation of routine reforms in China (24–26). Second, most evaluations primarily focus on general inpatient populations rather than older adults, even though multimorbidity and hospitalization needs are predominantly concentrated in later life (20). Third, many studies emphasize total inpatient expenditure, with insufficient attention given to patient-centered financial protection, particularly OOP spending, and to internal shifts in cost structure (12, 27–29). Fourth, average treatment effects may obscure clinically meaningful heterogeneity across different disease groups, procedures, and care pathways (15, 30).

To address these gaps, we evaluated the implementation of DRG among inpatients aged 60 years or older in a county-level referral hospital using an interrupted time series design. We analyzed total costs, OOP costs, drug costs, material costs, examination and laboratory costs, and LOS before and after the initiation of actual DRG settlement. This study contributes to the literature in three significant ways: by focusing on older adults, by emphasizing patient financial protection alongside total spending, and by assessing disease-group heterogeneity within a policy-relevant county hospital setting.

## Methods

### Study design

We employed a retrospective interrupted time series design to evaluate temporal changes in inpatient costs and resource utilization following the implementation of DRG-based payment reform among older adults. The interrupted time series analysis is a quasi-experimental method recommended for assessing population-level interventions introduced at a clearly defined time point when randomized allocation is impractical (31).

The study was conducted in a large tertiary Grade A general hospital located in a county-level city in eastern China. This hospital serves as the leading institution within the local integrated care system and acts as the primary regional referral center for inpatient, emergency, and critical care services. During the study period, it operated over 40 inpatient wards, maintained approximately 1,600 to 1,700 beds, and provided a comprehensive range of medical and surgical services. This context is relevant to policy discussions because county-level referral hospitals are expected to accommodate local inpatient demand, coordinate both upward and downward referrals, and spearhead the development of county medical alliances. Research on county medical alliances indicates substantial variation between counties in terms of implementation capacity, referral coordination, information integration, and the service capabilities of lead hospitals (32).

Local reform was implemented in a phased manner rather than instantaneously. According to local implementation records, a mixed payment system under global budget control, with DRG as the predominant component, was initiated in November 2021. All 11 secondary or higher-level medical institutions in the area commenced trial operations under the DRG system in January 2022, with actual DRG-based settlement beginning on July 1, 2022. Consequently, we used July 2022 as the intervention cutoff in the interrupted time series models, while acknowledging that coding changes, organizational preparation, and behavioral adaptation may have commenced earlier.

The broader policy environment further emphasizes the significance of this setting. In 2021, the National Healthcare Security Administration issued a three-year action plan to expedite DRG and Diagnosis-Related Group Payment (DIP) reform, aiming for full implementation across all planning areas by the end of 2024 and coverage of all eligible inpatient institutions by the end of 2025. In Province J, official reports indicated that by 2022, all designated inpatient institutions and all inpatient cases covered by DRG had uploaded standardized settlement data, achieving complete provincial coverage. Within this policy context, the transition to actual DRG settlement on July 1, 2022, serves as a meaningful operational benchmark for evaluating post-implementation changes, although it should not be interpreted as a perfectly isolated causal shock. To maintain institutional anonymity, the name of the hospital is not disclosed.

### Data source and study population

Data were obtained from the health insurance fund supervision system maintained by the local Healthcare Security Administration. This administrative database records insured inpatient admissions and includes information on patient demographics, diagnoses, procedures, payment types, hospitalization costs by category, length of stay, and discharge disposition. Diagnoses were coded in accordance with the International Statistical Classification of Diseases and Related Health Problems, Tenth Revision (ICD-10) (33). The study period spanned from July 1, 2021, to July 31, 2025, encompassing 12 months prior to and 37 months following the implementation of Diagnosis-Related Groups (DRG). The study population consisted of older adult inpatient admissions, with each admission treated as a separate observation.

Eligibility criteria were defined *a priori*. Admissions were included if they met all the following criteria: (1) hospitalization occurred within the study period; (2) the patient was aged 60 years or older at the time of admission; and (3) the admission belonged to the prespecified insured inpatient population captured in the administrative database and was eligible for evaluation under the local payment reform setting. A total of 157,012 admissions meeting the age and study-period criteria were screened. Of these, 2,159 (1.38%) non-local admissions, 108 (0.07%) admissions with missing principal diagnosis information, and one (<0.01%) admission with missing cost information were excluded. No other missing, invalid, or internally inconsistent records were identified. The final analytic sample comprised 154,744 admissions (98.56%).

Since the reform was implemented at the hospital and payment-system level rather than assigned to individual patients, all admissions meeting the prespecified eligibility criteria during the study period were included in the analysis.

### Outcome measures

We assessed six outcomes representing inpatient expenditure and resource utilization: total cost, out-of-pocket (OOP) cost, drug cost, material cost, examination and laboratory cost, and length of stay (LOS). Monetary outcomes were analyzed in nominal Chinese yuan (CNY) as recorded in the administrative database, with no inflation adjustment applied. Among these, the first five outcomes reflect various aspects of inpatient expenditure, while LOS serves as an indicator of inpatient resource utilization. Total cost is defined as the total expenditure incurred during an inpatient admission. OOP cost refers to the amount paid directly by the patient after insurance reimbursement. Drug cost, material cost, and examination and laboratory cost are defined as expenditures on medications, medical consumables and devices, and diagnostic and testing services, respectively. LOS is measured as the number of days from admission to discharge.

### Statistical analysis

Continuous variables are expressed as means with standard deviations (SD), while categorical variables are presented as counts and percentages (No. [%]). Differences between the pre- and post-DRG periods were analyzed using t-tests for continuous variables and chi-squared (χ^2^) tests for categorical variables. The primary analysis employed segmented regression interrupted time series models to estimate changes in inpatient expenditure and resource utilization following the implementation of Diagnosis-Related Groups (DRG). For each outcome, the models incorporated a continuous monthly time variable, an indicator for the post-intervention period, and an interaction term between time and the post-intervention period (34).

$$Y_{t}=\beta_{0}+\beta_{1}{\text{Time}}_{t}+\beta_{2}{\text{Post}}_{t}+\beta_{3}({\text{Time}}_{t}\times {\text{Post}}_{t})+\varepsilon_{t}$$

where $\beta_{0}$ denotes the baseline level, $\beta_{1}$ the pre-intervention monthly trend, $\beta_{2}$ the immediate level change at implementation, and $\beta_{3}$ the change in post-intervention trend relative to the pre-intervention trend. The intervention cutoff was July 2022.

Admission-level models were additionally adjusted for age, sex, disease group, type of surgery, payment type, and discharge disposition. As a complementary analysis, we estimated segmented regression models using monthly mean outcomes. This approach matched the time scale at which the policy was implemented and allowed direct assessment of serial correlation in the monthly series. Newey–West heteroskedasticity- and autocorrelation-consistent standard errors were used with a lag length of 3.

The two approaches address related but different aspects of the policy association. The admission-level model is weighted by the number of admissions in each month and allows covariate adjustment, whereas the monthly aggregated model gives each calendar month one observation and focuses on changes in hospital-level mean outcomes over time. The models also differ in their variance estimation. Their estimates were therefore interpreted as complementary rather than directly interchangeable, with greater emphasis placed on findings that were consistent across both approaches.

Robustness analyses included alternative symmetric bandwidths around the intervention cutoff, a placebo analysis utilizing a false cutoff 6 months prior to implementation, exclusion of the first 1 to 3 months following implementation, and additional analyses accounting for the COVID-19 policy-adjustment period (December 2022–February 2023) by including an indicator for this period and by excluding these months. Heterogeneity analyses were performed by disease group using stratified monthly aggregated interrupted time series models. All tests were two-sided, with *p* < 0.05 considered statistically significant.

## Results

Table 1 presents the characteristics of inpatient admissions during the pre-DRG and post-DRG periods. Among the total of 154,744 inpatient admissions, 35,037 occurred prior to the implementation of DRG, while 119,707 occurred afterward. The mean total cost, OOP cost, drug cost, and LOS were lower in the post-DRG period. Conversely, examination and laboratory costs increased, whereas material costs remained largely unchanged. Patient characteristics were generally comparable across both periods. The mean age exhibited minimal change, from 72.40 years in the pre-DRG period to 72.56 years in the post-DRG period, and the proportion of female admissions increased slightly, from 41.7 to 43.1%. Circulatory diseases, factors influencing health status and contact with health services, respiratory diseases, and digestive diseases constituted the most prevalent disease groups in both periods. The proportion of admissions involving surgery rose from 55.4% prior to DRG implementation to 61.8% afterward. The distribution of payment types also underwent a shift, with URRBMI increasing from 51.5 to 61.8%, while UEBMI decreased from 42.5 to 31.0%. The proportion of in-hospital deaths was 3.7% in the pre-DRG period and 4.6% in the post-DRG period.

**Table 1:** Characteristics of inpatient admissions before and after DRG implementation.

Variable	Overall (*N* = 154,744)	Pre-DRG (*N* = 35,037)	Post-DRG (*N* = 119,707)	*p* value
Total cost, mean (SD), CNY	15283.76 (22621.22)	15733.59 (23068.57)	15152.10 (22487.00)	<0.001
Out-of-pocket cost, mean (SD), CNY	5482.50 (10251.05)	6469.24 (12351.90)	5193.69 (9529.86)	<0.001
Drug cost, mean (SD), CNY	3245.82 (4993.54)	4280.13 (6282.54)	2943.09 (4502.97)	<0.001
Material cost, mean (SD), CNY	3656.03 (10561.34)	3837.19 (10719.74)	3603.01 (10513.99)	<0.001
Examination and laboratory cost, mean (SD), CNY	4598.56 (4537.12)	4133.03 (3026.17)	4734.82 (4883.48)	<0.001
Length of stay, mean (SD), days	8.05 (8.61)	8.59 (10.20)	7.89 (8.08)	<0.001
Age, mean (SD), years	72.52 (7.87)	72.40 (7.64)	72.56 (7.94)	<0.001
Sex, No. (%)				
Female	66,210 (42.8%)	14,607 (41.7%)	51,603 (43.1%)	<0.001
Male	88,534 (57.2%)	20,430 (58.3%)	68,104 (56.9%)	
Disease group, No. (%)				
Infectious and parasitic diseases	2,129 (1.4%)	424 (1.2%)	1,705 (1.4%)	<0.001
Neoplasms	13,993 (9.0%)	2,916 (8.3%)	11,077 (9.3%)	
Blood and immune disorders	1,476 (1.0%)	367 (1.0%)	1,109 (0.9%)	
Endocrine and metabolic diseases	6,147 (4.0%)	1,268 (3.6%)	4,879 (4.1%)	
Mental and behavioral disorders	411 (0.3%)	41 (0.1%)	370 (0.3%)	
Nervous system diseases	3,436 (2.2%)	956 (2.7%)	2,480 (2.1%)	
Eye diseases	5,486 (3.5%)	1,124 (3.2%)	4,362 (3.6%)	
Ear diseases	925 (0.6%)	246 (0.7%)	679 (0.6%)	
Circulatory system diseases	30,582 (19.8%)	7,182 (20.5%)	23,400 (19.5%)	
Respiratory diseases	16,187 (10.5%)	3,367 (9.6%)	12,820 (10.7%)	
Digestive diseases	14,940 (9.7%)	3,192 (9.1%)	11,748 (9.8%)	
Skin/subcutaneous diseases	653 (0.4%)	184 (0.5%)	469 (0.4%)	
Musculoskeletal diseases	5,139 (3.3%)	1,153 (3.3%)	3,986 (3.3%)	
Genitourinary diseases	8,239 (5.3%)	1,666 (4.8%)	6,573 (5.5%)	
Congenital anomalies	233 (0.2%)	59 (0.2%)	174 (0.1%)	
Symptoms and abnormal findings	3,865 (2.5%)	821 (2.3%)	3,044 (2.5%)	
Injury, poisoning and external	12,004 (7.8%)	2,653 (7.6%)	9,351 (7.8%)	
Factors influencing health	28,899 (18.7%)	7,418 (21.2%)	21,481 (17.9%)	
Surgery, No. (%)				
No	61,323 (39.6%)	15,619 (44.6%)	45,704 (38.2%)	<0.001
Yes	93,421 (60.4%)	19,418 (55.4%)	74,003 (61.8%)	
Payment type, No. (%)				
URRBMI	91,993 (59.4%)	18,052 (51.5%)	73,941 (61.8%)	<0.001
UEBMI	51,952 (33.6%)	14,897 (42.5%)	37,055 (31.0%)	
NRCMS	68 (0.0%)	38 (0.1%)	30 (0.0%)	
Full Public	816 (0.5%)	101 (0.3%)	715 (0.6%)	
Self-paid	8,373 (5.4%)	1,948 (5.6%)	6,425 (5.4%)	
Other	1,542 (1.0%)	1 (0.0%)	1,541 (1.3%)	
Discharge disposition, No. (%)				
Death	6,798 (4.4%)	1,279 (3.7%)	5,519 (4.6%)	<0.001
Survived	147,946 (95.6%)	33,758 (96.3%)	114,188 (95.4%)	

(1) DRG, diagnosis-related groups; OOP, out-of-pocket; SD, standard deviation; LOS, length of stay; URRBMI, Urban and Rural Resident Basic Medical Insurance; UEBMI, Urban Employee Basic Medical Insurance; NRCMS, New Rural Cooperative Medical Scheme.

(2) Data are mean (SD) or No. (%). *p* values were calculated using t tests for continuous variables and χ^2^ tests for categorical variables.

### Interrupted time series estimates

Table 2 presents the interrupted time series estimates at both the individual and monthly aggregated levels, while Figure 1 illustrates the corresponding monthly trajectories. Across both modeling approaches, the most consistent findings were a reduction in OOP costs and an increase in LOS following July 2022. In the individual-level models, OOP costs exhibited a significant immediate decline along with a more negative trend post-intervention, whereas LOS demonstrated both an immediate increase and a positive change in slope after the intervention. In contrast, no robust changes in level or slope were observed for total cost or most cost components at the individual level. In the monthly aggregated models, out-of-pocket costs and length of stay displayed directionally similar patterns. Supplementary analysis of OOP expenditure as a proportion of total hospitalization costs yielded consistent findings, showing a significant immediate decline and a more negative post-intervention trend in both modeling approaches (Supplementary Table S1). Total costs and material costs experienced immediate declines in the monthly aggregated models; however, these findings were not consistently replicated in the individual-level models. Examination and laboratory costs did not exhibit a clear policy-related shift in the primary ITS models.

**Table 2:** Interrupted time series estimates of DRG implementation at the individual and monthly aggregated levels.

	(1)	(2)	(3)	(4)	(5)	(6)
	Total cost	OOP	Drug cost	Material	Exam and lab	LOS
Panel A. Individual-level interrupted time series models						
Baseline level, $\beta_{0}$	6259.779 (1028.481)^***^	1729.889 (393.989)^***^	3037.321 (298.716)^***^	−404.579 (448.109)	3673.470 (254.876)^***^	0.049 (0.440)
Pre-intervention monthly trend, $\beta_{1}$	−99.097 (39.958)^**^	50.984 (28.068)^*^	−75.410 (11.631)^***^	−21.416 (20.171)	19.648 (13.391)	−0.133 (0.012)^***^
Immediate level change at cutoff, $\beta_{2}$	−84.683 (345.292)	−1360.617 (307.895)^***^	102.887 (119.547)	−130.285 (178.250)	45.776 (154.904)	0.831 (0.180)^***^
Post-intervention slope change, $\beta_{3}$	34.844 (40.797)	−106.705 (29.356)^***^	14.719 (11.997)	−8.168 (20.503)	−4.574 (15.245)	0.077 (0.014)^***^
Observations	154744	154744	154744	154744	154744	154744
Panel B. Monthly aggregated interrupted time series models						
Baseline level, $\beta_{0}$	16006.758 (215.728)^***^	5987.019 (210.541)^***^	4723.310 (103.149)^***^	3750.340 (127.179)^***^	3986.782 (128.199)^***^	9.434 (0.084)^***^
Pre-intervention monthly trend, $\beta_{1}$	−43.953 (26.732)	71.932 (32.285)^**^	−66.341 (14.452)^***^	11.088 (18.550)	21.666 (15.593)	−0.127 (0.013)^***^
Immediate level change at cutoff, $\beta_{2}$	−851.064 (268.584)^***^	−1623.711 (476.923)^***^	47.369 (131.423)	−616.850 (192.233)^***^	−4.338 (213.031)	0.683 (0.255)^**^
Post-intervention slope change, $\beta_{3}$	75.090 (30.132)^**^	−77.871 (31.522)^**^	13.135 (15.909)	6.753 (19.420)	3.769 (20.503)	0.096 (0.014)^***^
Observations, months	49	49	49	49	49	49

Standard errors in parentheses.

(1) Panel A reports individual-level interrupted time series estimates with standard errors clustered at the calendar-month level. Panel B reports monthly aggregated estimates with Newey-West heteroskedasticity- and autocorrelation-consistent standard errors (lag = 3), selected by formula: floor(4*(N/100)^(2/9)).

(2) The intervention cutoff was July 2022.

(3) $\beta_{0}$ denotes the intercept: in Panel A, the predicted outcome at study onset for the reference covariate profile; in Panel B, the fitted mean outcome in the first study month. $\beta_{1}$ denotes the pre-intervention monthly trend, $\beta_{2}$ the immediate level change at the DRG cutoff, and $\beta_{3}$ the post-intervention slope change.

(4) Individual-level models additionally control for age, sex, disease group, surgery, payment type, and discharge disposition.

(5) OOP indicates out-of-pocket cost; LOS, length of stay.

^*^p < 0.10, ^**^p < 0.05, ^***^p < 0.01.

**Figure 1:**
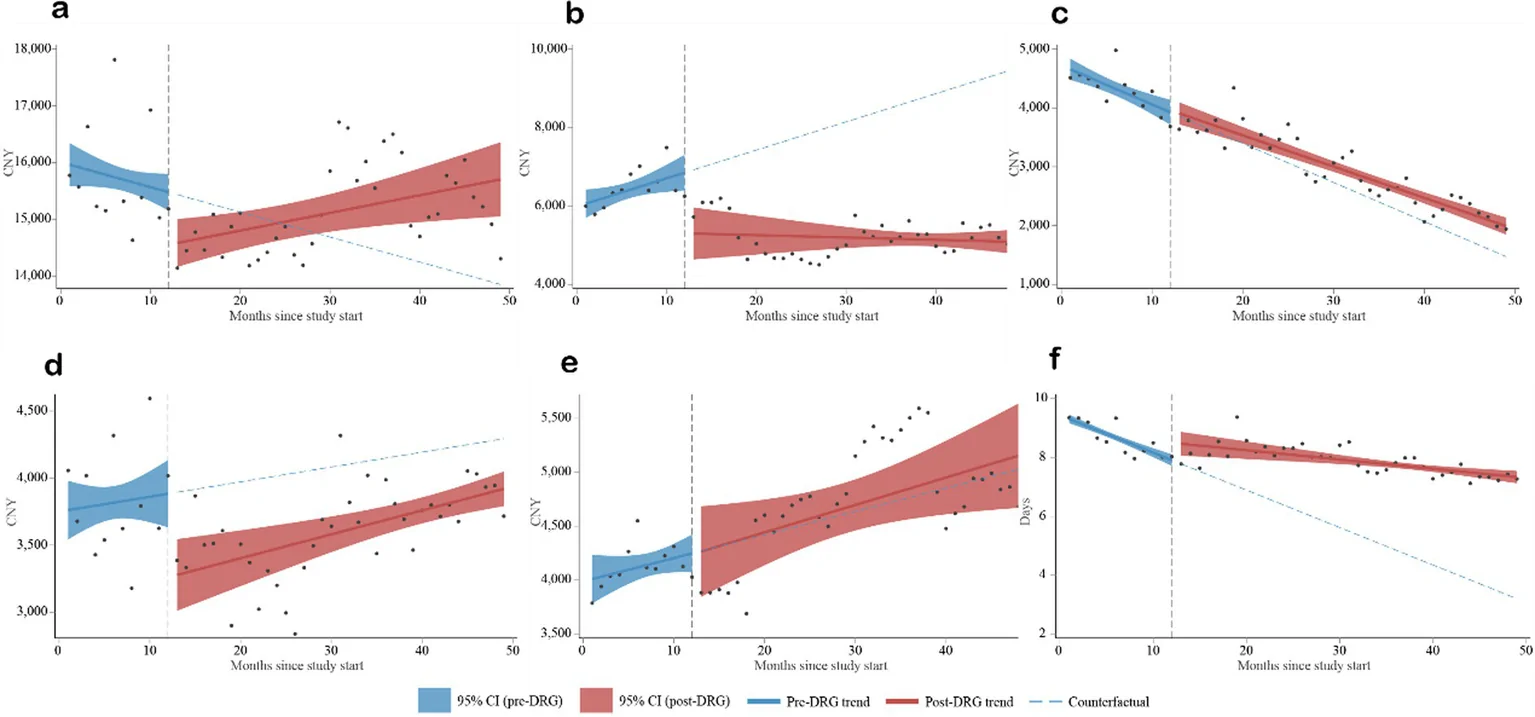
Monthly aggregated interrupted time series of inpatient outcomes before and after DRG implementation. (1) Panels **(a–f)** present total cost, out-of-pocket cost, drug cost, material cost, examination and laboratory cost, and length of stay, respectively. (2) The vertical dashed line marks the DRG implementation point in July 2022. (3) The solid blue and red lines indicate the fitted pre-DRG and post-DRG trends, respectively; the shaded areas indicate 95% CIs; and the dashed blue line indicates the counterfactual projection based on the pre-DRG trend.

### Robustness analyses

Table 3 presents robustness analyses regarding the immediate level change following the implementation of the DRG. Throughout the analysis windows, the estimated immediate changes in total costs and material costs remained consistently negative across alternative bandwidths. Furthermore, the estimated immediate decline in OOP costs was negative in all specifications and achieved statistical significance in the broader windows. Conversely, drug costs did not exhibit a clear immediate change across the various bandwidths. In the placebo analysis, estimates at the false cutoff were generally not statistically significant, with the notable exception of examination and laboratory costs. Similar findings were observed after excluding either the initial 1 to 3 months post-implementation or the COVID-19 period: total costs, OOP costs, and material costs remained lower, while LOS remained elevated.

**Table 3:** Robustness analyses of immediate level change ( $\beta_{2}$ ) following DRG implementation.

Specification	(1)	(2)	(3)	(4)	(5)	(6)
	Total cost	OOP	Drug cost	Material	Exam and lab	LOS
Panel A. Bandwidth sensitivity analysis						
±6 months	−1298.746 (505.844)^*^	−384.792 (282.553)	114.981 (73.362)	−851.796 (265.732)^*^	−160.135 (71.634)	−0.300 (0.168)
±9 months	−793.355 (345.548)^*^	−345.497 (248.220)	−12.866 (127.018)	−484.614 (174.249)^*^	−325.728 (118.995)^*^	−0.035 (0.149)
±12 months	−851.593 (260.235)^**^	−753.717 (278.619)^*^	−67.248 (106.884)	−403.834 (170.352)^*^	−499.899 (146.968)^**^	0.233 (0.209)
±18 months	−1023.664 (225.179)^***^	−1070.953 (372.512)^**^	27.525 (137.742)	−473.051 (178.765)^*^	−446.144 (132.537)^**^	0.396 (0.261)
±24 months	−1335.625 (295.233)^***^	−1439.797 (479.357)^**^	54.662 (133.167)	−635.547 (197.715)^**^	−477.271 (129.254)^***^	0.580 (0.288)
±36 months	−928.779 (241.408)^***^	−1622.391 (462.128)^**^	44.576 (126.754)	−628.170 (185.823)^**^	−62.001 (186.088)	0.680 (0.247)^**^
Panel B. Placebo test						
Placebo cutoff, January 2022	−1514.822 (812.618)	61.903 (197.515)	−186.249 (230.727)	−358.000 (318.502)	−413.071 (99.664)^**^	−0.597 (0.308)
Panel C. Excluding transition period						
Exclude 1-month transition	−770.687 (277.330)^**^	−1676.802 (482.535)^**^	24.311 (125.373)	−611.075 (191.713)^**^	63.465 (207.216)	0.728 (0.221)^**^
Exclude 2-month transition	−717.017 (292.798)^*^	−1777.307 (476.883)^***^	−18.938 (123.409)	−598.442 (186.427)^**^	138.473 (207.146)	0.736 (0.218)^**^
Exclude 3-month transition	−695.740 (311.607)^*^	−1888.261 (447.827)^***^	−44.205 (121.507)	−647.032 (175.799)^***^	218.172 (196.323)	0.803 (0.162)^***^
Panel D. Excluding the COVID-19 period (*N* = 46 months)						
Immediate level change	−847.601 (291.005)^**^	−1536.410 (477.694)^**^	−17.195 (118.171)	−604.228 (196.864)^**^	39.095 (227.543)	0.575(0.228)^*^

Standard errors are reported in parentheses.

(1) All entries report estimates of $\beta_{2}$ , from monthly aggregated interrupted time series models estimated with Newey–West heteroskedasticity- and autocorrelation-consistent standard errors.

(2) Panel A re-estimates the model using alternative symmetric time windows centered on the intervention cutoff. Panel B reports placebo estimates based on pre-DRG observations only, using an artificial cutoff 6 months before the true policy date. Panel C re-estimates the model after sequentially excluding the first 1–3 months following DRG implementation to assess sensitivity to short-term transition effects. Panel D re-estimates the model excluding observations from the COVID-19 period, defined as December 2022 through February 2023, to assess the robustness of the results to pandemic-related disruptions.

^*^p < 0.10, ^**^p < 0.05, ^***^p < 0.01.

### Subgroup analysis

Figure 2 presents exploratory estimates of immediate level changes stratified by disease group following the implementation of DRG. Notably, total costs exhibited immediate reductions predominantly in nervous system and digestive diseases, while an increase was recorded in eye diseases. In terms of OOP costs, negative immediate changes were observed across several disease categories, including neoplasms, nervous system diseases, digestive diseases, circulatory system diseases, respiratory diseases, and genitourinary diseases. Drug costs decreased in the categories of digestive diseases, nervous system diseases, and congenital anomalies, whereas material costs declined in digestive diseases as well as endocrine and metabolic diseases. Examination and laboratory costs displayed smaller and less consistent changes across the various disease groups. LOS increased in multiple disease categories, including blood and immune disorders, circulatory system diseases, respiratory diseases, symptoms and abnormal findings, and factors influencing health status and contact with health services; however, it decreased in congenital anomalies and nervous system diseases. Detailed results are presented in Supplementary Table S2.

**Figure 2:**
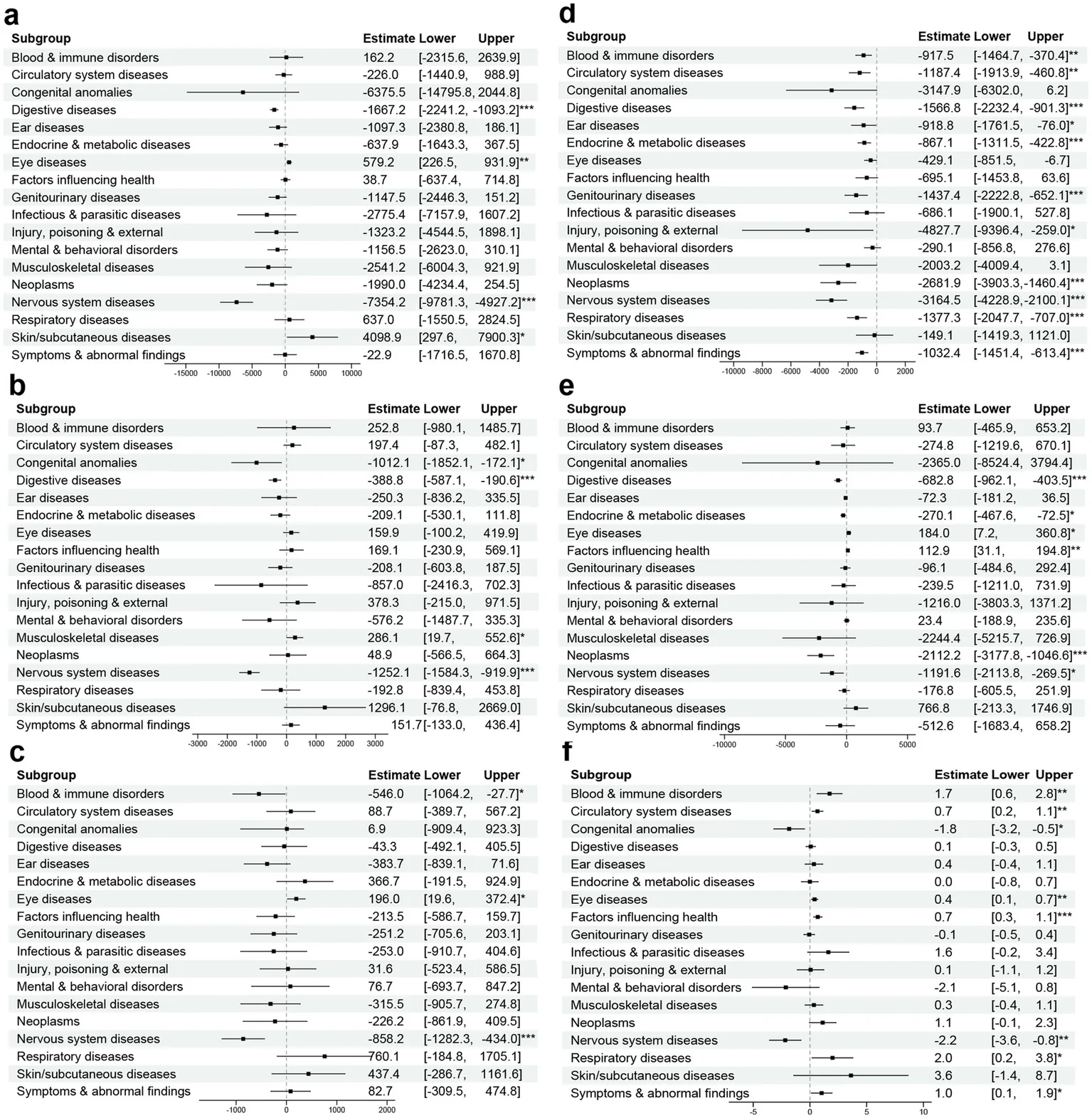
Immediate level changes in inpatient outcomes after DRG implementation across disease groups. (1) Forest plots of the immediate level change parameter ( $\beta_{2}$ ) from monthly aggregated interrupted time series models stratified by disease group. (2) Panel **(a)** shows total cost; panel **(b)**, drug cost; panel **(c)**, examination and laboratory cost; panel **(d)**, out-of-pocket cost; panel **(e)**, material cost; and panel **(f)**, length of stay. (3) Squares indicate point estimates and horizontal lines indicate 95% CIs.

## Discussion

This study examined temporal changes in inpatient spending and resource use following DRG implementation among older adults in a county-level referral hospital. Three findings were most notable. First, OOP spending declined consistently across the main analyses. Second, LOS increased rather than decreased after implementation. Third, evidence for changes in total cost and cost composition was less consistent across model specifications and levels of analysis.

The reduction in OOP spending may reflect changes in hospital service use under prospective payment. DRG payment weakens the link between hospital revenue and the volume of individual services and encourages hospitals to control unnecessary or high-cost care (35, 36). OOP spending can decrease if hospitals reduce services or items with higher patient cost-sharing, even when total cost changes little. This pattern has also been reported in some Chinese DRG settings (16, 37), although the effect on patient financial burden differs across payment systems (18). The finding is important for older adults because multimorbidity and repeated hospitalization are common in this population. A reduction in OOP spending per admission may therefore reduce cumulative financial burden (38, 39).

The cost-component results suggest changes in the use of specific resources rather than a uniform reduction in spending. Drugs and medical materials can often be standardized, substituted, or reduced under DRG payment. Examinations and laboratory tests may be less flexible when they are needed for diagnosis and monitoring. This is relevant to older patients with several coexisting conditions, who may still require repeated testing during hospitalization. Reductions in one cost category can therefore occur without a similar reduction in total cost. Previous studies have also found that DRG reform can change the composition of inpatient spending (19, 28). This provides a possible explanation for the clearer changes in some cost components than in total expenditure.

The LOS result is closely related to the care needs of older patients. LOS increased at the time of implementation relative to the expected trend, but continued to decline afterward at a slower rate. Older inpatients often have multimorbidity, lower functional reserve, and greater needs for monitoring, rehabilitation, and discharge planning. Discharge may depend on control of the main disease, management of coexisting conditions, functional recovery, family support, and arrangements for follow-up care (40). Reducing drugs, materials, or other services does not necessarily shorten the time required for safe discharge. Previous evidence from China also shows that the effect of case-based payment on LOS can differ by age, with older groups showing different post-reform trends (41). These factors may explain why our LOS findings differ from studies of broader inpatient populations that reported shorter stays after DRG reform (6, 27, 30, 42).

The disease-group results can also be understood from differences in clinical pathways and resource use. OOP reductions were more evident in neoplasms and circulatory, digestive, respiratory, nervous system, and genitourinary diseases. These conditions often involve repeated treatment, drug use, diagnostic testing, or relatively structured treatment pathways, which may provide more room to adjust service use under DRG payment. Total cost reductions in nervous system and digestive diseases may reflect greater scope for changes in treatment and resource use (30). The increase in eye-disease costs may reflect differences in procedure and material use. LOS increased mainly in some circulatory and respiratory conditions, where older patients may need longer monitoring and stabilization because of acute illness and coexisting diseases (42–45). Differences in treatment intensity, procedure use, and discharge needs can therefore produce different responses across disease groups.

The county-level setting also affects how DRG incentives are translated into hospital practice. The study hospital provides complex inpatient care and coordinates referrals with lower-level facilities. For older patients, discharge and transfer can depend on local referral capacity and the availability of continued care. Studies of county medical alliances have shown that referral coordination, service capacity, and organizational integration affect hospital performance (25, 46, 47). These factors can limit the extent to which payment reform alone reduces LOS or total cost. The findings show the value of assessing DRG reform in older adults using patient financial burden, cost structure, and resource use together, particularly in county-level referral hospitals.

### Implications

The findings have several practical implications for DRG implementation in county-level hospitals. Monitoring should include OOP spending, cost components, and LOS rather than total cost alone. A fall in OOP spending should be assessed together with changes in LOS and quality to determine whether lower patient payments are accompanied by efficient care. For older patients, hospitals should strengthen discharge planning and coordination with lower-level facilities, especially for circulatory and respiratory diseases in which LOS showed greater increases. Disease-specific monitoring may also help identify cost shifting between drugs, materials, and diagnostic services. Health insurance authorities should therefore combine payment indicators with readmission, mortality, complications, ICU transfer, and case-mix measures when evaluating DRG performance.

### Strengths and limitations

This study possesses several strengths. It utilized a longitudinal administrative dataset encompassing a substantial number of admissions among older adults, examined multiple expenditure outcomes alongside LOS, and employed both admission-level and monthly aggregated interrupted time series analyses. Additionally, it explored heterogeneity across various disease groups. However, several limitations must also be acknowledged. First, this study is an observational interrupted time series analysis without a comparison group, which necessitates that the findings be interpreted as temporal associations rather than definitive causal effects. Second, concurrent changes cannot be disregarded, including alterations in reimbursement rules, auditing intensity, procurement reforms, shifts in inpatient demand post-COVID, hospital management responses, and variations in case-mix or referral flow. Third, the database lacked key quality and severity-related indicators such as readmissions, complications, ICU transfers, DRG weight, case-mix index, frailty, functional status, or post-discharge outcomes. This limitation is significant, as an extended LOS may indicate a greater need for care, delayed discharge, conservative provider behavior, or unmeasured severity, rather than inefficiency alone. Fourth, the single-hospital design constrains generalizability, although the hospital serves as a policy-relevant institution within a county-level alliance.

## Conclusion

The DRG-based payment reform implemented in this county-level referral hospital was associated with a reduction in OOP spending among older inpatients. However, the changes in total costs and cost composition were less consistent, alongside an increase in LOS. Disease-group analyses further indicate that these associations were not uniform, reflecting clinically heterogeneous responses to the reform. These findings advocate for a comprehensive evaluation framework for DRG implementation in county hospitals, one that integrates considerations of financial protection, resource utilization, quality, and disease-specific heterogeneity.

## Data Availability

The datasets used and/or analyzed during the current study are available from the corresponding author on reasonable request. Requests to access these datasets should be directed to Jiacheng Zou, 220244334@seu.edu.cn.
