# Supplementary material for: Association of DRG-based payment reform with inpatient costs and resource use among older adults: an interrupted time series study in a county-level referral hospital in China
Source: Front Public Health. 2026 Sep 17;14:1953054. doi: 10.3389/fpubh.2026.1953054 (PMC13627011; doi:10.3389/fpubh.2026.1953054)
Supplement: Supplementary file 1 [file Table_1.docx]

**Supplementary Table S1. Additional interrupted time series estimates of DRG implementation at the individual and monthly aggregated levels**

|  | **Outcome** |
| --- | --- |
| **Panel A. Individual-level interrupted time series model** | |
| Baseline level, $\beta_{0}$ | 38.553(1.658)*** |
| Pre-intervention monthly trend, $\beta_{1}$ | 0.730(0.138)*** |
| Immediate level change at cutoff, *β₂* | -8.754(1.674)*** |
| Post-intervention slope change, *β₃* | -0.867(0.146)*** |
| Observations | 154744 |
| **Panel B. Monthly aggregated interrupted time series model** | |
| Baseline level, $\beta_{0}$ | 35.540(0.995)*** |
| Pre-intervention monthly trend, $\beta_{1}$ | 0.759(0.191)*** |
| Immediate level change at cutoff, *β₂* | -8.089(2.471)*** |
| Post-intervention slope change, *β₃* | -0.807(0.184)*** |
| Observations, months | 49 |

Notes: Standard errors in parentheses

(1) Panel A reports individual-level interrupted time series estimates with standard errors clustered at the calendar-month level. Panel B reports monthly aggregated estimates with Newey-West heteroskedasticity- and autocorrelation-consistent standard errors (lag = 3), selected by formula: floor(4*(N/100)^(2/9)).

(2) The intervention cutoff was July 2022.

(3) $\beta_{0}$ denotes the intercept: in Panel A, the predicted outcome at study onset for the reference covariate profile; in Panel B, the fitted mean outcome in the first study month. $\beta_{1}$ denotes the pre-intervention monthly trend, $\beta_{2}$ the immediate level change at the DRG cutoff, and $\beta_{3}$ the post-intervention slope change.

(4) Individual-level models additionally control for age, sex, disease group, surgery, payment type, and discharge disposition.

(5) OOP indicates out-of-pocket cost; LOS, length of stay.

^*^ *p* < 0.10, ^**^ *p* < 0.05, ^***^ *p* < 0.01

**Supplementary Table S2. Disease-group-specific immediate level changes following DRG implementation**

Panel A. Total hospitalization costs

| **Disease group** | **Admissions, n** | ***β₂*** | **SE** | **95% CI lower** | **95% CI upper** | **P value** |
| --- | --- | --- | --- | --- | --- | --- |
| Blood & immune disorders | 1,476 | 162.2 | 1264.2 | -2315.6 | 2639.9 | 0.898 |
| Circulatory system diseases | 30,582 | -226.0 | 619.9 | -1440.9 | 988.9 | 0.717 |
| Congenital anomalies | 233 | -6375.5 | 4296.1 | -14795.8 | 2044.8 | 0.145 |
| Digestive diseases | 14,940 | -1667.2 | 292.8 | -2241.2 | -1093.2 | <0.001 |
| Ear diseases | 925 | -1097.3 | 654.8 | -2380.8 | 186.1 | 0.101 |
| Endocrine & metabolic diseases | 6,147 | -637.9 | 513.0 | -1643.3 | 367.5 | 0.220 |
| Eye diseases | 5,486 | 579.2 | 179.9 | 226.5 | 931.9 | 0.002 |
| Factors influencing health | 28,899 | 38.7 | 344.9 | -637.4 | 714.8 | 0.911 |
| Genitourinary diseases | 8,239 | -1147.5 | 662.6 | -2446.3 | 151.2 | 0.090 |
| Infectious & parasitic diseases | 2,129 | -2775.4 | 2236.0 | -7157.9 | 1607.2 | 0.221 |
| Injury, poisoning & external | 12,004 | -1323.2 | 1643.5 | -4544.5 | 1898.1 | 0.425 |
| Mental & behavioral disorders | 411 | -1156.5 | 748.3 | -2623.0 | 310.1 | 0.130 |
| Musculoskeletal diseases | 5,139 | -2541.2 | 1766.9 | -6004.3 | 921.9 | 0.157 |
| Neoplasms | 13,993 | -1990.0 | 1145.1 | -4234.4 | 254.5 | 0.089 |
| Nervous system diseases | 3,436 | -7354.2 | 1238.3 | -9781.3 | -4927.2 | <0.001 |
| Respiratory diseases | 16,187 | 637.0 | 1116.1 | -1550.5 | 2824.5 | 0.571 |
| Skin/subcutaneous diseases | 653 | 4098.9 | 1939.5 | 297.6 | 7900.3 | 0.040 |
| Symptoms & abnormal findings | 3,865 | -22.9 | 864.1 | -1716.5 | 1670.8 | 0.979 |

Panel B. Drug costs

| **Disease group** | **Admissions, n** | ***β₂*** | **SE** | **95% CI lower** | **95% CI upper** | **P value** |
| --- | --- | --- | --- | --- | --- | --- |
| Blood & immune disorders | 1,476 | 252.8 | 629.0 | -980.1 | 1485.7 | 0.690 |
| Circulatory system diseases | 30,582 | 197.4 | 145.3 | -87.3 | 482.1 | 0.181 |
| Congenital anomalies | 233 | -1012.1 | 428.6 | -1852.1 | -172.1 | 0.023 |
| Digestive diseases | 14,940 | -388.8 | 101.1 | -587.1 | -190.6 | <0.001 |
| Ear diseases | 925 | -250.3 | 298.9 | -836.2 | 335.5 | 0.407 |
| Endocrine & metabolic diseases | 6,147 | -209.1 | 163.8 | -530.1 | 111.8 | 0.208 |
| Eye diseases | 5,486 | 159.9 | 132.7 | -100.2 | 419.9 | 0.235 |
| Factors influencing health | 28,899 | 169.1 | 204.1 | -230.9 | 569.1 | 0.412 |
| Genitourinary diseases | 8,239 | -208.1 | 201.8 | -603.8 | 187.5 | 0.308 |
| Infectious & parasitic diseases | 2,129 | -857.0 | 795.6 | -2416.3 | 702.3 | 0.287 |
| Injury, poisoning & external | 12,004 | 378.3 | 302.7 | -215.0 | 971.5 | 0.218 |
| Mental & behavioral disorders | 411 | -576.2 | 465.1 | -1487.7 | 335.3 | 0.222 |
| Musculoskeletal diseases | 5,139 | 286.1 | 135.9 | 19.7 | 552.6 | 0.041 |
| Neoplasms | 13,993 | 48.9 | 314.0 | -566.5 | 664.3 | 0.877 |
| Nervous system diseases | 3,436 | -1252.1 | 169.5 | -1584.3 | -919.9 | <0.001 |
| Respiratory diseases | 16,187 | -192.8 | 329.9 | -839.4 | 453.8 | 0.562 |
| Skin/subcutaneous diseases | 653 | 1296.1 | 700.4 | -76.8 | 2669.0 | 0.071 |
| Symptoms & abnormal findings | 3,865 | 151.7 | 145.3 | -133.0 | 436.4 | 0.302 |

Panel C. Examination and laboratory costs

| **Disease group** | **Admissions, n** | ***β₂*** | **SE** | **95% CI lower** | **95% CI upper** | **P value** |
| --- | --- | --- | --- | --- | --- | --- |
| Blood & immune disorders | 1,476 | -546.0 | 264.4 | -1064.2 | -27.7 | 0.045 |
| Circulatory system diseases | 30,582 | 88.7 | 244.1 | -389.7 | 567.2 | 0.718 |
| Congenital anomalies | 233 | 6.9 | 467.5 | -909.4 | 923.3 | 0.988 |
| Digestive diseases | 14,940 | -43.3 | 229.0 | -492.1 | 405.5 | 0.851 |
| Ear diseases | 925 | -383.7 | 232.3 | -839.1 | 71.6 | 0.106 |
| Endocrine & metabolic diseases | 6,147 | 366.7 | 284.8 | -191.5 | 924.9 | 0.204 |
| Eye diseases | 5,486 | 196.0 | 90.0 | 19.6 | 372.4 | 0.035 |
| Factors influencing health | 28,899 | -213.5 | 190.4 | -586.7 | 159.7 | 0.268 |
| Genitourinary diseases | 8,239 | -251.2 | 231.8 | -705.6 | 203.1 | 0.284 |
| Infectious & parasitic diseases | 2,129 | -253.0 | 335.5 | -910.7 | 404.6 | 0.455 |
| Injury, poisoning & external | 12,004 | 31.6 | 283.2 | -523.4 | 586.5 | 0.912 |
| Mental & behavioral disorders | 411 | 76.7 | 393.1 | -693.7 | 847.2 | 0.846 |
| Musculoskeletal diseases | 5,139 | -315.5 | 301.2 | -905.7 | 274.8 | 0.300 |
| Neoplasms | 13,993 | -226.2 | 324.3 | -861.9 | 409.5 | 0.489 |
| Nervous system diseases | 3,436 | -858.2 | 216.4 | -1282.3 | -434.0 | <0.001 |
| Respiratory diseases | 16,187 | 760.1 | 482.1 | -184.8 | 1705.1 | 0.122 |
| Skin/subcutaneous diseases | 653 | 437.4 | 369.5 | -286.7 | 1161.6 | 0.243 |
| Symptoms & abnormal findings | 3,865 | 82.7 | 200.1 | -309.5 | 474.8 | 0.681 |

Panel D. Out-of-pocket costs

| **Disease group** | **Admissions, n** | ***β₂*** | **SE** | **95% CI lower** | **95% CI upper** | **P value** |
| --- | --- | --- | --- | --- | --- | --- |
| Blood & immune disorders | 1,476 | -917.5 | 279.2 | -1464.7 | -370.4 | 0.002 |
| Circulatory system diseases | 30,582 | -1187.4 | 370.7 | -1913.9 | -460.8 | 0.002 |
| Congenital anomalies | 233 | -3147.9 | 1609.2 | -6302.0 | 6.2 | 0.057 |
| Digestive diseases | 14,940 | -1566.8 | 339.6 | -2232.4 | -901.3 | <0.001 |
| Ear diseases | 925 | -918.8 | 430.0 | -1761.5 | -76.0 | 0.038 |
| Endocrine & metabolic diseases | 6,147 | -867.1 | 226.7 | -1311.5 | -422.8 | <0.001 |
| Eye diseases | 5,486 | -429.1 | 215.5 | -851.5 | -6.7 | 0.053 |
| Factors influencing health | 28,899 | -695.1 | 387.1 | -1453.8 | 63.6 | 0.079 |
| Genitourinary diseases | 8,239 | -1437.4 | 400.7 | -2222.8 | -652.1 | 0.001 |
| Infectious & parasitic diseases | 2,129 | -686.1 | 619.4 | -1900.1 | 527.8 | 0.274 |
| Injury, poisoning & external | 12,004 | -4827.7 | 2331.0 | -9396.4 | -259.0 | 0.044 |
| Mental & behavioral disorders | 411 | -290.1 | 289.1 | -856.8 | 276.6 | 0.321 |
| Musculoskeletal diseases | 5,139 | -2003.2 | 1023.6 | -4009.4 | 3.1 | 0.057 |
| Neoplasms | 13,993 | -2681.9 | 623.2 | -3903.3 | -1460.4 | <0.001 |
| Nervous system diseases | 3,436 | -3164.5 | 543.1 | -4228.9 | -2100.1 | <0.001 |
| Respiratory diseases | 16,187 | -1377.3 | 342.0 | -2047.7 | -707.0 | <0.001 |
| Skin/subcutaneous diseases | 653 | -149.1 | 648.0 | -1419.3 | 1121.0 | 0.819 |
| Symptoms & abnormal findings | 3,865 | -1032.4 | 213.8 | -1451.4 | -613.4 | <0.001 |

Panel E. Material costs

| **Disease group** | **Admissions, n** | ***β₂*** | **SE** | **95% CI lower** | **95% CI upper** | **P value** |
| --- | --- | --- | --- | --- | --- | --- |
| Blood & immune disorders | 1,476 | 93.7 | 285.5 | -465.9 | 653.2 | 0.744 |
| Circulatory system diseases | 30,582 | -274.8 | 482.1 | -1219.6 | 670.1 | 0.572 |
| Congenital anomalies | 233 | -2365.0 | 3142.6 | -8524.4 | 3794.4 | 0.456 |
| Digestive diseases | 14,940 | -682.8 | 142.5 | -962.1 | -403.5 | <0.001 |
| Ear diseases | 925 | -72.3 | 55.5 | -181.2 | 36.5 | 0.199 |
| Endocrine & metabolic diseases | 6,147 | -270.1 | 100.8 | -467.6 | -72.5 | 0.010 |
| Eye diseases | 5,486 | 184.0 | 90.2 | 7.2 | 360.8 | 0.047 |
| Factors influencing health | 28,899 | 112.9 | 41.8 | 31.1 | 194.8 | 0.010 |
| Genitourinary diseases | 8,239 | -96.1 | 198.2 | -484.6 | 292.4 | 0.630 |
| Infectious & parasitic diseases | 2,129 | -239.5 | 495.6 | -1211.0 | 731.9 | 0.631 |
| Injury, poisoning & external | 12,004 | -1216.0 | 1320.0 | -3803.3 | 1371.2 | 0.362 |
| Mental & behavioral disorders | 411 | 23.4 | 108.3 | -188.9 | 235.6 | 0.830 |
| Musculoskeletal diseases | 5,139 | -2244.4 | 1516.0 | -5215.7 | 726.9 | 0.146 |
| Neoplasms | 13,993 | -2112.2 | 543.7 | -3177.8 | -1046.6 | <0.001 |
| Nervous system diseases | 3,436 | -1191.6 | 470.5 | -2113.8 | -269.5 | 0.015 |
| Respiratory diseases | 16,187 | -176.8 | 218.7 | -605.5 | 251.9 | 0.423 |
| Skin/subcutaneous diseases | 653 | 766.8 | 500.0 | -213.3 | 1746.9 | 0.132 |
| Symptoms & abnormal findings | 3,865 | -512.6 | 597.3 | -1683.4 | 658.2 | 0.395 |

Panel F. Length of stay

| **Disease group** | **Admissions, n** | ***β₂*** | **SE** | **95% CI lower** | **95% CI upper** | **P value** |
| --- | --- | --- | --- | --- | --- | --- |
| Blood & immune disorders | 1,476 | 1.7 | 0.6 | 0.6 | 2.8 | 0.003 |
| Circulatory system diseases | 30,582 | 0.7 | 0.2 | 0.2 | 1.1 | 0.008 |
| Congenital anomalies | 233 | -1.8 | 0.7 | -3.2 | -0.5 | 0.011 |
| Digestive diseases | 14,940 | 0.1 | 0.2 | -0.3 | 0.5 | 0.740 |
| Ear diseases | 925 | 0.4 | 0.4 | -0.4 | 1.1 | 0.381 |
| Endocrine & metabolic diseases | 6,147 | 0.0 | 0.4 | -0.8 | 0.7 | 0.972 |
| Eye diseases | 5,486 | 0.4 | 0.1 | 0.1 | 0.7 | 0.007 |
| Factors influencing health | 28,899 | 0.7 | 0.2 | 0.3 | 1.1 | 0.001 |
| Genitourinary diseases | 8,239 | -0.1 | 0.2 | -0.5 | 0.4 | 0.800 |
| Infectious & parasitic diseases | 2,129 | 1.6 | 0.9 | -0.2 | 3.4 | 0.088 |
| Injury, poisoning & external | 12,004 | 0.1 | 0.6 | -1.1 | 1.2 | 0.928 |
| Mental & behavioral disorders | 411 | -2.1 | 1.5 | -5.1 | 0.8 | 0.163 |
| Musculoskeletal diseases | 5,139 | 0.3 | 0.4 | -0.4 | 1.1 | 0.388 |
| Neoplasms | 13,993 | 1.1 | 0.6 | -0.1 | 2.3 | 0.068 |
| Nervous system diseases | 3,436 | -2.2 | 0.7 | -3.6 | -0.8 | 0.003 |
| Respiratory diseases | 16,187 | 2.0 | 0.9 | 0.2 | 3.8 | 0.035 |
| Skin/subcutaneous diseases | 653 | 3.6 | 2.6 | -1.4 | 8.7 | 0.167 |
| Symptoms & abnormal findings | 3,865 | 1.0 | 0.5 | 0.1 | 1.9 | 0.031 |

Notes: *β*₂ denotes the estimated immediate level change at the DRG implementation cutoff. SE denotes standard error; CI, confidence interval; DRG, diagnosis-related group. P values are two-sided as reported in the subgroup models. Disease-group analyses are exploratory.
